# 636. Epidemiology and Outcome Among the Elderly Hospitalized Patients with Respiratory Syncytial Virus Infections

**DOI:** 10.1093/ofid/ofad500.702

**Published:** 2023-11-27

**Authors:** Ashish Bhargava, Susan M Szpunar, Ambreen Malik, Hussein Tehaili, Mamta Sharma

**Affiliations:** Ascension St. John Hospital; Wayne State University School of Medicine, Grosse Pointe Woods, Michigan; Ascension St. John Hospital, MI; Ascension St John Hospital, Warren, Michigan; Ascension St. John Hospital, MI; Ascension St John Hospital & Medical Center, Grosse Pointe Woods, MI. 48236, Michigan

## Abstract

**Background:**

Elderly patients with congestive heart failure (CHF), chronic lung diseases (CLD) and immunocompromised conditions are considered a high-risk category for severe respiratory syncytial virus (RSV) infections. Our study aimed to identify RSV disease epidemiology and its outcomes among the elderly hospitalized patients with and without high-risk conditions (HRC).

**Methods:**

We conducted a multicenter historical cohort study of adult patients for laboratory-confirmed RSV-related diseases in Ascension hospitals in southeast Michigan between January 2017 and December 2021. Patients were identified using ICD 10 codes for RSV diseases. Patients with HRC were those elderly patients with CHF, CLD and immune-compromised conditions. Data were analyzed using SPSS v. 29.0.

**Results:**

Among 240 patients, 31.3% (75) were without HRC whereas 68.7% (165) had at least one HRC. The mean (sd) age of the patients without HRC was 81.9 + 8.9 years, 47 (62.7%) were female, 52 (69.3%) were white and mean body mass index (BMI) was 28.9 + 8.3 years. The mean (sd) age of the patients with HRC was 76.6 + 7.7 years, 111 (67.3%) were female, 125 (75.8%) were white and BMI was 29.8 + 9.0 years. Major comorbidities among patients without HRC compared to those with HRC were hypertension (77.3% vs 69.1%), cerebrovascular condition (25.3 vs 10.9%), diabetes (18% vs 37.6%), dementia (21.3% vs 8.5%), and chronic kidney disease (CKD) (16% vs 21.2%). Current smokers were 30.7% vs 66.1% among patients without HRC compared to those with HRC. On admission, median (IQR) qSOFA scores were 0 (0-2) vs 1 (0-3) among patients without HRC compared to those with HRC. Lower respiratory tract infections (LRTI) were 32% vs 43%, acute kidney injury (22.7% vs 24.2%), liver injury (1.3% vs 3%), mechanical ventilation (6.7% vs 9.1%), pressors (1.3% vs 1.8%), and ICU requirement (10.7% vs 15.2%) among patients without HRC compared to those with HRC. In-hospital mortality were 1.3% vs 5.5% among patients without HRC compared to those with HRC.

**Conclusion:**

In our study, elderly patients with HRC were younger, smokers with higher incidence of diabetics, CKD, higher qSOFA on presentation with higher LRTIs, and other complications including mortality. Targeted risk mitigation strategies can help reduce disease burden and cost associated with RSV infections.

**Disclosures:**

**All Authors**: No reported disclosures

